# Chemical Composition, Antioxidant, Anti-Inflammatory and Anti-Proliferative Activities of Essential Oils of Plants from Burkina Faso

**DOI:** 10.1371/journal.pone.0092122

**Published:** 2014-03-24

**Authors:** Bagora Bayala, Imaël Henri Nestor Bassole, Charlemagne Gnoula, Roger Nebie, Albert Yonli, Laurent Morel, Gilles Figueredo, Jean-Baptiste Nikiema, Jean-Marc A. Lobaccaro, Jacques Simpore

**Affiliations:** 1 Centre de Recherche Biomoléculaire Pietro Annigoni (CERBA), Laboratoire de Biologie et Génétique (LABIOGENE), Centre Médical Saint Camille, Ouagadougou, Université de Ouagadougou, Ouagadougou, Burkina Faso; 2 Clermont Université, Université Blaise Pascal, Génétique Reproduction et Développement (GReD), Clermont-Ferrand, France; 3 Centre National de la Recherche Scientifique (CNRS), Unité Mixte de Recherche (UMR) 6293, GReD, Aubière, France; 4 Institut National de la Santé et de la Recherche Médicale (INSERM), UMR 1103, GReD, Aubière, France; 5 Centre de Recherche en Nutrition Humaine d'Auvergne, Clermont-Ferrand, France; 6 Laboratoire de Biochimie Alimentaire, Enzymologie, Biotechnologies industrielles et Bioinformatique (BAEBIB), Unité de Formation et de Recherche en Sciences de la Vie et de la Terre (UFR-SVT), Université de Ouagadougou, Ouagadougou, Burkina Faso; 7 Laboratoire de Pharmacologie, de Toxicologie et de Chimie Thérapeutique, Unité de Formation et de Recherche en Sciences de la Santé (UFR-SDS), Université de Ouagadougou, Ouagadougou, Burkina Faso; 8 Institut de Recherche en Sciences Appliquées et Techniques, Département des Substances Naturelles, Ouagadougou, Burkina Faso; 9 LEXVA Analytique, Biopole Clermont-Limagne, Saint-Beauzire, France; 10 Centre Médical Saint Camille de Ouagadougou, Ouagadougou, Burkina Faso; Institut de Génomique Fonctionnelle de Lyon, France

## Abstract

This research highlights the chemical composition, antioxidant, anti-inflammatory and anti-proliferative activities of essential oils from leaves of *Ocimum basilicum*, *Ocimum americanum*, *Hyptis spicigera*, *Lippia multiflora*, *Ageratum conyzoides*, *Eucalyptus camaldulensis* and *Zingiber officinale*. Essential oils were analyzed by gas chromatography–mass spectrometry and gas chromatography–flame ionization detector. Major constituents were α-terpineol (59.78%) and β-caryophyllene (10.54%) for *Ocimum basilicum*; 1, 8-cineol (31.22%), camphor (12.730%), α-pinene (6.87%) and trans α-bergamotene (5.32%) for *Ocimum americanum*; β-caryophyllene (21%), α-pinene (20.11%), sabinene (10.26%), β-pinene (9.22%) and α-phellandrene (7.03%) for *Hyptis spicigera*; p-cymene (25.27%), β-caryophyllene (12.70%), thymol (11.88), γ-terpinene (9.17%) and thymyle acetate (7.64%) for *Lippia multiflora*; precocene (82.10%)for *Ageratum conyzoides*; eucalyptol (59.55%), α-pinene (9.17%) and limonene (8.76%) for *Eucalyptus camaldulensis*; arcurcumene (16.67%), camphene (12.70%), zingiberene (8.40%), β-bisabolene (7.83%) and β-sesquiphellandrène (5.34%) for *Zingiber officinale*. Antioxidant activities were examined using 1,1-diphenyl-2-picryl-hydrazyl (DPPH) and 2,2′-azinobis-(3-ethylbenzothiazoline-6-sulfonic acid (ABTS) methods. *O. basilicum* and *L. multiflora* exhibited the highest antioxidant activity in DPPH and ABTS tests, respectively. Anti-inflammatory properties were evaluated by measuring the inhibition of lipoxygenase activity and essential oil of *Z. officinale* was the most active. Anti-proliferative effect was assayed by the measurement of MTT on LNCaP and PC-3 prostate cancer cell lines, and SF-763 and SF-767 glioblastoma cell lines. Essential oils from *A. conyzoides* and *L. multiflora* were the most active on LNCaP and PC-3 cell lines, respectively. The SF-767 glioblastoma cell line was the most sensitive to *O. basilicum* and *L. multiflora* EOs while essential oil of *A. conyzoides* showed the highest activity on SF-763 cells. Altogether these results justify the use of these plants in traditional medicine in Burkina Faso and open a new field of investigation in the characterization of the molecules involved in anti-proliferative processes.

## Introduction

Representing 7.6 million deaths worldwide, or approximately 13% of deaths, cancer is the second cause of mortality [1]. Cancer is nowadays case of major death in the world particularly in the low income countries and middle-incomes. Those numbers of cancer mortality could increase by 50% to reach 15 million by 2030 worldwide [1]. In Africa, in 2008, 682,000 people have been affected by cancer. The mortality was 572,402 during the same period. Epidemiological studies provide 1.2 million new cases of cancer in Africa by 2030 with more than 970,000 dead if adequate preventive measures are not taken quickly. The most common types of cancer in Africa are women cervical cancer of the uterus, breast and primary liver cancers, and men prostate cancer and Kaposi's sarcoma; even though epidemiological data regarding cancer in Sub-Saharan Africa are scarce. In Burkina Faso, the few statistics show that the annual incidence of cancer from January 1986 to December 2006 in the three laboratories of anatomy and pathological cytology of Ouagadougou was 200 cases per year [2]. This extremely low rate was mainly explained by the very low participation of the population in screening of cancers. The most frequent cancers for men are skin cancer (11.37%), followed by non-Hodgkin lymphoma (9.80%) and prostate cancer (9.69%) while breast (23.81%) and uterine neck cancers (22.99%) are the most frequent for women [2].

Plants are a potential source of drug discovery and development of cancer chemoprevention [3], [4]. They could thus provide a hope for finding anticancer molecules available and efficient for the treatment of persons with cancer. In fact, many cytotoxic molecules, which are of plant origin, are widely used in chemotherapy [5]. Burkina Faso is a country with many unknown plant whose compounds could be used in medicine [6]. Essential oils (EOs) are volatile, complex compounds characterized by a strong odor and are formed by aromatic plants as secondary metabolites. They have been widely used for bactericidal, virucidal, fungicidal, antiparasitical, insecticidal, anticancer, antioxidant, antidiabetic, cardiovascular, and cosmetic and food applications [7], [8]. Among the various plants with putative pharmacological properties, *Ocimum basilicum* Linnæus, *Ocimum americanum* Linnæus, *Hyptis spicigera* Lamarck, *Lippia multiflora* Moldenk, *Ageratum conyzoides* Linnæus, *Eucalyptus camaldulensis* Dehnhardt and *Zingiber officinale* Roscoe are common in Burkina Faso. Leaves and leaf stems of these plants are widely used in traditional medicine in Burkina Faso among others to treat angina, swellings, wounds, scorpion and snake bites, malaria, hemorrhoids, rheumatism, fevers, nervous dyspepsia, rheumatism, uterus diseases, jaundice [9] and are also used as antioxidant, anti-inflammatory and fungicide [9]. The plants used in the treatment of certain inflammatory and oxidative diseases may have anticancer effects. Indeed, there is a relationship between the production of reactive oxygen species (ROS) to the origin of oxidation and inflammation that can lead to cancer [10]. In fact, initial experiments on the role of ROS in tumor initiation have assumed that oxidative stress acts as a DNA-damaging agent, effectively increasing the mutation rate within cells and thus promoting oncogenic transformation [10]. However, recent studies have revealed that in addition to inducing genomic instability, ROS can specifically activate signaling pathways and thus contribute to tumor development through the regulation of cellular proliferation, angiogenesis, and metastasis [11]. Chronic inflammation has been linked to various steps involved in carcinogenesis, including cellular transformation, promotion, survival, proliferation, invasion, angiogenesis, and metastasis [12], [13]. Several human chronic disease states including cancer have been associated with oxidative stress produced through either an increased free radical generation and/or a decreased antioxidant level in the target cells and tissues [14], [15].

From previous studies carried on biological activities on EOs, *O. americanum* has insecticidal [16], antimicrobial [17], [18] and antibacterial [19] activities. Likewise EO of *O. basilicum* has larvicidal [20], antimicrobial [21], antileishmanial [22], antifungal [23], anticancer [24] activities. EO extracted from *H. spicigera* has shown insecticidal [16], [25], antimicrobial, anticancer and insecticidal [26], gastroprotective and ulcer healing effects [27]. EO from *L. multiflora* is analgesic, antipyretic anti-inflammatory [28], and antimicrobial [21]. EO extracted from *A. conyzoides L.* is antifungal, suppressor of the potent mycotoxin aflatoxin B1 [29], insecticidal [30] and anti-inflammatory [31]. *E. camaldulensis* has larvicidal [32], insecticidal [33], and acaricidal [34] activities. *Z. officinale* has been shown to be antifungal [35], antiradical [36] and larvicidal [37]; besides its EO also presents inhibitory effects on leukocyte migration [38], antioxidant activity [39], antibacterial and anti-cancer activity [40]. At last, EO of *E. camaldulensis* has larvicidal [32], insecticidal [33] and acaricidal activities [34].

The aim of the present study was to investigate chemical compositions and chemotypes, antioxidant, anti-inflammatory and antiproliferative activities of EOs from these seven plants of Burkina Faso *in vitro* as well as on cell cultures.

## Materials and Methods

### Plant material

*O. basilicum*, *O. americanum*, *H. spicigera*, *L. multiflora*, *A. conyzoides*, *E. camaldulensis* and *Z. officinale* were collected during June 2010 in Gampéla, 25 km East from Ouagadougou (Latitude N 12 27.075, Longitude W 1 20.161; GPS location: 12.451244,-1.336023). Plants, which are not endangered or protected, were identified by Dr. Jeanne Millogo–Rasolodimby (Plant Biology and Ecology Laboratory, Ouagadougou University) and a voucher specimen was deposited under numbers 15941, 15939, 15942, 15938, 13162, 15943 and 15944 for *O. basilicum*, *O. americanum*, *H. spicigera*, *L. multiflora*, *A. conyzoides*, *E. camaldulensis* and *Z. officinale* respectively in the herbarium of the Plant Biology and Ecology Laboratory. No specific permission was required for this plant collection.

### Essential oils (EOs)

Fractions of each dried plant material (200 g) were submitted to hydrodistillation using an alembic/Clevenger-type apparatus for 3 h as previously described [41]. Anhydrous sodium sulfate was used to remove water after extraction. EOs were stored in airtight containers in a refrigerator at 4°C until GC-FID and GC/MS analyses and biological tests. EOs were diluted in hexane (1/30, v/v) for GC/FID analysis.

### Gas chromatography–flame ionization detector (GC/FID) analysis

Gas chromatography was performed on an Agilent gas chromatograph Model 6890 (Agilent, Palo Alto, Ca), equipped with a DB5 MS column (30 m×0.25 mm, 0.25 µm film thickness). Hydrogen was used as carrier gas (flow 1.0 ml/min). Oven temperature program was from 50°C (5 min) to 300°C at 5°C/min, 5 min post run at 300°C. Sample (1 µL) was injected in split mode (1∶60); injector and detector temperatures were 280 and 300°C, respectively.

### Gas chromatography–mass spectrometry (GC/MS) analysis

EOs were analyzed on an Agilent gas chromatograph Model 7890, coupled to a Agilent MS model 5975, equipped with a DB5 MS column (20 m×0.20 mm, 0.20 µm film thickness), programming from 50°C (5 min) to 300°C at 8°C/min, 5 min hold. Helium was used as carrier gas (average flow 1.0 ml/min). Oven temperature program was from 50°C (3.2 min) to 300°C at 8°C/min, 5 min post run at 300°C. Sample (1 µL) was injected in split mode (1∶150); injector and detector temperature were at 250°C and 280°C respectively. The MS working in electron impact mode at 70 eV; electron multiplier, 1500 V; ion source temperature, 230°C; mass spectra data were acquired in the scan mode in *m/z* range 33–450.

### Identification of components

**Figure 1:**
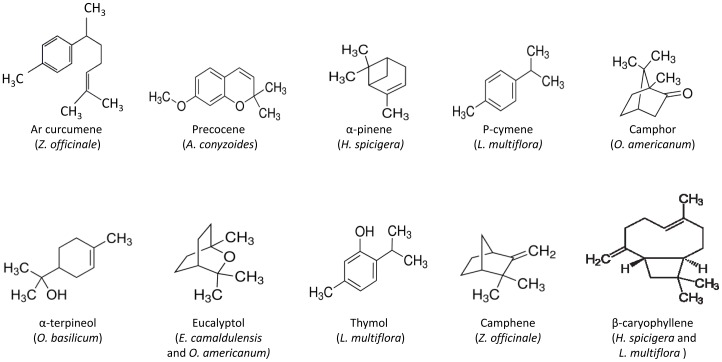
Chemical structures of the major compounds found in the analyzed essential oils.

Oil constituents were identified by comparison of their retention indices with those of the literature, determined in relation to a homologous series of n-alkanes (C8–C32) under the same operating conditions. Further identification was made by comparison of their mass spectra with those stored in NIST library [42] or with mass spectra from literature [43]. Component relative percentages were calculated based on GC peak areas without using correction factors. The major identified compounds are indicated on Figure 1.

### DPPH radical scavenging assay

DPPH (Sigma-Aldrich, L'Ile d'Abeau, France) radical scavenging activity was measured as described by Velasquez [20]. Briefly, 0.5 mL of EO (8 mg/mL in methanol) was added to 1 mL of DPPH solution (20 mg/mL in methanol) freshly prepared. After shaking, the mixture was incubated for 15 min in darkness at room temperature and then absorbance was measured at 517 nm against a blank (mixture without EO). Quercetin (Sigma-Aldrich) was used as positive control. Inhibition percentage of free DPPH radicals (I %) was calculated following the formula: I (%) = (1−A Sample/A Blank)×100, A_blank_ and A_sample_ being the absorbance of the blank and sample reactions respectively.

### ABTS^+^ radical cation decolorisation assay

The spectrophotometric analysis of ABTS^+^ scavenging activity was determined according to the method Re et al. [44]. Preparation of ABTS^+.^solution was done by dissolving 10 mg of ABTS in 2.6 ml of distilled water. Then, 1.7212 mg of potassium persulfate was added and the mixture is kept in the dark at room temperature for a maximum of 12 hours. The mixture was then diluted with ethanol in order to obtain an absorbance of 0.70±0.02 to 734 nm. In 96-well plates, 50 µl of test sample solution of essential oil (8 mg/mL) were added to 200 µl of ABTS^+^ solution freshly prepared. The same process was performed for quercetin (1 mg/mL) used as a positive control. Wholes are protected from light for 15 min and the concentration is read at 734 nm in a spectrophotometer against a standard curve with 6-hydroxy-2,5,7,8-tetramethylchroman-2-carboxylic acid (Trolox, Sigma-Aldrich). Concentration compounds having a reducing effect on the radical cation ABTS^+^ (antioxidant) is expressed in micromoles Trolox equivalent per gram of EO (µmolET/g) using the following formula: C = (c×D)/Ci. C, concentration of antioxidant compounds in μMET/g; c, concentration of sample read; D, dilution factor; Ci, concentration of stock solution.

### Anti-inflammatory capacity

Lipoxygenase (EC 1.13.11.12) type I-B inhibiting activity was essayed spectrophotometricaly as described by [45] with minor modifications. Briefly 100 µL of the enzyme solution (at the final concentration of 200 U/mL) was prepared in boric acid buffer (0.2 M; pH 9.0), mixed with 25 µL of extract solution (8 mg/mL in boric acid buffer) and then incubated at room temperature for 3 min. Reaction was subsequently initiated by the addition of substrate solution (linoleic acid, 250 µM), and the velocity was recorded for 90 seconds at 234 nm. Boric acid buffer was used as negative control (activity of enzyme without extract solution). The percentage of lipoxygenase inhibition was calculated according to the following equation: Inhibition (%) = (Vo_control_−Vo_sample_)×100/Vo _control_. Vo_control_ is the activity of enzyme in absence of extract solution, and Vo_sample_ is the activity of the enzyme in the presence of the analyzed EOs.

### Cell culture

Two human prostate cancer cell lines were used: LNCaP, an androgen responsive cell line with a low metastatic potential derived from a lymph node metastasis [46], and PC-3, an androgen insensitive cell line with a high metastatic potential derived from bone metastasis [47]. Two human glioblastoma cell lines with disparate radiation sensitivity were used, SF-763 and SF-767. Cells were grown at 37°C in a humidified chamber with 5% CO_2_ as monolayer adherent cultures in 75 cm^2^ tissue culture flasks, in a medium supplemented with 10% fetal calf serum (FCS, Biowest, Nuaillé, France), 1% penicillin and 1% streptomycin (Invitrogen, Oslo, Norway). LNCaP and PC-3 Cells were maintained in RPMI-1640 (Invitrogen). SF-767 and SF-763 cells were maintained in DMEM (Invitrogen).

### Cytotoxicity assay

Stock solution of 10 µl/ml of EOs was prepared in 1% DMSO (Sigma-Aldrich) in complete medium. Global cell growth was assessed using the colorimetric MTT (3[4,5-dimethylthiazol-2-yl]-diphenyltetrazolium bromide) assay (Sigma-Aldrich). Cells were incubated for 24 hours in 96-well plates (50,000 cells/ml) before incubation with EOs. Cell proliferation test is based on the ability of living cells to reduce MTT (yellow) into its metabolite blue formazan (violet). After 72 h incubation with or without EO, the number of living cells is directly proportional to the intensity of the violet color measured quantitatively by spectrophotometry using a microplate reader type Bio-Rad 11885 at 490 nm. Each experimental condition was analyzed in quadruplicate, with three experiments for each EO. Growth inhibition was calculated as follow: % growth inhibition = 100−(OD_test sample_−OD_blank_/OD_control_−OD_blank_)×100.

### Statistical analysis

All *in vitro* experiments were done in quadruplicate and each data point represents the average of at least three independent experiments. All data are reported as the mean ± SD. Data were analyzed by 1-way analysis of variance followed by the Tukey multiple-comparison test. Analyses were done by using XLSTAT7.1 software. A P value less than 0.05 was used as the criterion for statistical significance.

## Results and Discussion

### Composition analysis of the various essential oils

**Figure 2:**
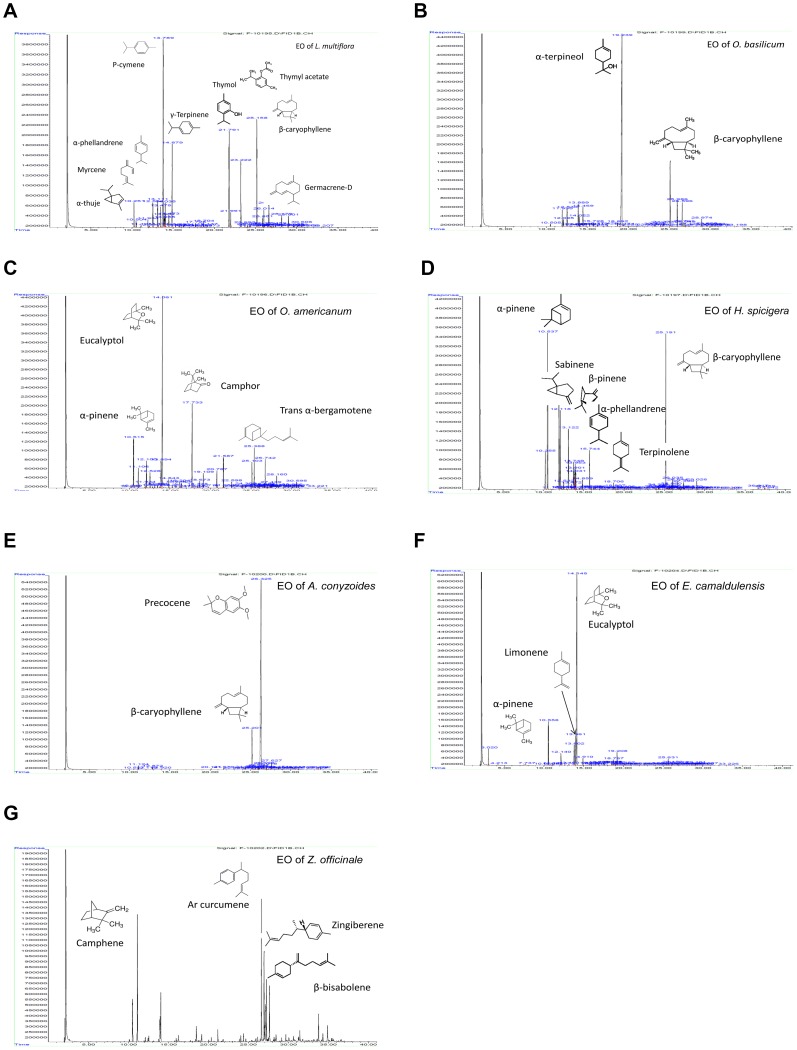
Chromatograms of the various EOs with their major identified compounds. A) *O. basilicum*; B) *O. americanum*; C) *H. spicigera*; D) *L. multiflora*; E) *A. conizoides*; F) *A. calmadulensis*; G) *Z. officinale*.

Steam distillation, followed by GC/MS and GC/FID analyses allowed determining the composition of EOs of the seven plants from Burkina Faso. Chromatograms with the identified peaks as well as the chemical structures of the major compounds are shown in Figure 2. Percentage composition of the oil components are listed in Table 1. Briefly, EOs mainly contain a complex mixture of monoterpene hydrocarbons, sesquiterpene hydrocarbons and oxygen containing mono- and sesquiterpenes (Figure 1). Monoterpenes hydrocarbons were dominant in the EOs of *E. camaldulensis, L. multiflora, H. spicigera* and *O. americanum*. Sesquiterpene hydrocarbons were the most abundant in the EO of *Z. officinale*. Oxygenated terpenes were the most dominant constituents of the EOs of *A. conyzoides* and *O. basilicum*. The EO of *L. multiflora* and *O. americanum* also contained relative percentage of oxygenated terpenes, 25.10% and 23.94%, respectively.

**Table 1:** Chemical composition (in %) of essential oils of the seven aromatic plants tested.

Compounds	rt	*A. conyzoides*	*E. camaldulensis*	*L. multiflora*	*H. spicigera*	*O. americanum*	*O. basilicum*	*Z. officinale*
α-thujene	10.249	—	—	2.63	4.37	0.13	—	—
α-pinene	10.505	0.10	9.17	0.68	20.11	6.87	0.39	4.14
Camphene	11.106	0.80	0.19	0.23	0.09	2.77	—	12.69
Sabinene	11.935	0.07	—	0.86	10.26	0.65	2.58	—
β-pinene	12.095	—	2.49	0.14	9.22	3.71	1.08	0.46
Myrcene	12.530	—	0.15	2.67	0.81	2.04	2.71	0.60
α-phellandrene	13.100	—	0.32	2.97	7.03	0.39	—	—
α-terpinene	13.46	0.45	—	2.18	0.61	0.40	0.11	—
P-cymene	13.740	—	4.73	25.27	3.05	0.21	0.14	0.28
Limonene	13.889	0.10	8.76	1.21	2.33	4.66	3.69	2.08
β-phellandrene	13.953	—	—	—	2.69	—	—	2.46
Eucalyptol	14.022	—	59.55	3.37	1.81	31.22	1.46	4.79
(E)-β-ocimene	14.469	—	—	1.23	0.13	—	2.86	—
γ-Terpinene	14.854	—	1.39	9.17	1.09	1.13	0.16	—
Terpinolene	15.728	—	0.21	0.17	4.43	0.65	0.60	—
Camphor	17.733	—	—	0.46	—	12.73		—
Borneol	18.432	—	0.46	—	—	0.13	—	1.55
Terpinene-4-ol	18.692	—	1.14	0.55	0.75	0.92	0.63	—
α-terpineol	19.239	—	2.65	0.25	—	2.08	59.78	0.78
Piperitone	20.787	—	0.09	—	0.08	2.44	—	—
Geranial	21.181	—	—	—	—	—	—	1.21
bornyl Acetate	21.587	0.23	—	—	0.05	3.96	—	—
Thymol	21.791	—	—	11.88	—	—	—	—
Carvacrol	21.991	—	—	1.67	—	—	—	—
Thymyle Acetate	23.222	—	—	7.64	—	—	—	—
β-caryophyllene	25.116	8.49	—	12.70	21.00	3.55	10.54	—
Trans α-bergamotene	25.388	0.12	—	—	—	5.32	—	—
Aromadendrene	25.630	—	1.40	—	—	—	—	—
α-humulene	25.986	0.64	—	1.89	1.14	0.25	3.90	—
Precocene	26.324	82.09	—	—	—	—	—	—
Germacrene-D	26.596	1.30	—	2.50	1.00	0.24	3.72	—
Ar curcumene	26.643	—	—	—	—	—	—	16.67
(Z, E)-α-farnesene	26.742	—	—	—	—	3.67	—	—
Zingiberene	26.957	—	—	—	—	—	—	8.40
γ-bulgarene	27.113	—	—	—	—	—	—	3.79
(E,E)-α-farnesene	27.143	—	—	—	—	—	—	2.58
β-bisabolène	27.263	—	—	—	—	—	—	7.83
β-sesquiphellandrene	27.627	1.42	—	—	—	—	—	5.33
Elemol	28.16	—	—	1.25	—	1.60	—	0.44
Caryophyllene oxide	28.974	0.15	—	1.40	0.98	0.08	1.29	—
TOTAL		98.79	97.43	99.23	96.72	98.90	99.45	83.30
Monoterpene hydrocarbon		1.52	86.96	52.78	68.03	54.83	15.78	27.5
Sesquiterpene hydrocarbon		11.97	1.4	17.09	23.14	13.03	18.16	44.6
Oxygenated terpenes		82.47	4.34	25.10	1.86	23.94	61.7	3.98

rt, retention time (min).

A total of 29 compounds were identified in the EO of *O. basilicum* (Figure 2A). Two major compounds were identified: α-terpineol (59.78%) and β-caryophyllene (10.54%). The minor compounds were α-humulene (3.90%), Germacrene-D (3.72%), Limonene (3.69%), (E)-β-ocimene (2.86%), Myrcene (2.71%), Eucalyptol (1.46%), Caryophyllene oxide (1.29%) and β-pinene (1.08%). On the basis of the oil composition, seven chemotypes of *O. basilicum* essential oil have been described: (1) high-linalool, (2) linalool/eugenol, (3) methyl chavicol without linalool, (4) methyl chavicol/linalool, (5) methyl eugenol/linalool, (6) methyl cinnamate/linalool and (7) bergamotene chemotypes [48]. The terpineol α/β caryophyllene chemotype of the EO in leaves of *O. basilicum* of our analysis (Table 1) has not been reported yet. This is not surprising as the composition of EO depends on genetics, age, season and/or environment of the plant [49].

In the EO of *O. americanum*, 44 compounds were identified (Figure 2B); 1, 8-cineol (31.22%), Camphor (12.730%), α-pinene (6.87%) and trans α-bergamotene (5.32%) were the most abundant compounds. Minor components were Limonene (4.66%), bornyl Acetate (3.96%), β-pinene (3.71%), farnesene (3.67%), β-caryophyllene (3.55%), Camphene (2.77%), Myrcene (2.04%), α-terpineol (2.08%), Piperitone (2.44%), (Z, E)-α-Elemol (1.60%), Terpinene (1.13%). This composition was different from that reported for Indian *O. americanum* [50]. Conversely, our data corroborate the data published by Djibo et al. [51].

EO of *H. spicigera* pointed out 39 compounds (Figure 2C); major compounds appear such as β-caryophyllene (21%), α-pinene (20.11%), Sabinene (10.26%), β-pinene (9.22%), α-phellandrene (7.03%); minors are Terpinolene (4.43%), α-thujene (4.37%), P-cymene (3.05%), β-phellandrene (2.69%), Limonene (2.33%), Eucalyptol (1.81%), α-humulene (1.14%), γ-Terpinene (1.09%) and Germacrene-D (1.00%). Similar composition has been reported by McNeil et al. [26].

EO of *L. multiflora* contains 42 compounds (Figure 2D); among them, the major molecules were p-cymene (25.27%), β-caryophyllene (12.70%), thymol (11.88), γ-terpinene (9.17%), thymyle acetate (7.64%). The minor components were Eucalyptol (3.37%), α-phellandrene (2.97%), α-thujene (2.63%), Myrcene (2.67%), Germacrene-D (2.50%), α-terpinene (2.18%), α-humulene (1.89%), Carvacrol (1.67%), Caryophyllene oxide (1.40%), (E)-β-ocimene (1.23%), Elemol (1.25%) and Limonene (1.21%). This composition was different from that previously reported [21]

Twenty three compounds were identified in the EO of *A. conyzoides* (Figure 2E). Precocene (82.10%) was the most dominant followed by caryophyllene (8.50%). Minor components were β-sesquiphellandrene (1.42%) and Germacrene-D (1.30%). This composition is comparable to that reported by De Melo et al. [52] and Nogueira et al. [29]. Abdelkader and Lockwood [53] described precocene I, germacrene D, β-caryophyllene and precocene II as the main constituents.

In the EO of *E. camaldulensis* (Figure 2F), 39 compounds were identified, among which Eucalyptol (59.55%), α-pinene (9.17%) and limonene (8.76%) were the most dominant. The minor components were P-cymene (4.73%), α-terpineol (2.65%), β-pinene (2.49%), Aromadendrene (1.40%), γ-Terpinene (1.39%) and Terpinene-4-ol (1.14%). A similar composition has been reported by Da Cruz et al. [54] but differs from the chemotype isolated by Herzi et al. [55].

Essential oil of *Z. officinale* contained 35 compounds (Figure 2G). The major compounds are arcurcumene (16.67%), camphene (12.70%), zingiberene (8.40%), β-bisabolene (7.83%), β-sesquiphellandrène (5.34%). These results were comparable to those reported by Nogueira de Melo et al. [38] which found arcurcumene (59%), β-myrcene (14%), 1,8-cineole (8%), citral (7.5%), and zingiberene (7.5%) as main compounds. Eucalyptol (4.79%), α-pinene (4.14%), β-phellandrene (2.46%), Limonene (2.08%), Borneol (1.55%) and Geranial (1.21%) were the minor components of this EO.

### Antioxidant activity of the essential oils

An antioxidant can be defined as any substance capable of competing with other oxidizable substrates at relatively low concentrations and delay or prevent the oxidation of these substrates. The DPPH radical-scavenging activities of the seven EOs and of references are shown in Table 2. EO *of O. basilicum* showed the best ability to scavenge DPPH^+^ radical created *in vitro* with a percentage of inhibition of 55.67% for a concentration of 8 mg/mL while *L. multiflora* exhibited the highest capability to reduce ABTS^+^ (1.02 µmolET/g) followed by *O. basilicum*. Besides, *E. camaldulensis*, *L. multiflora* and *H. spicigera* respectively also presented interesting antioxidant activities. For some EOs, results differed between DPPH and ABTS methods. These variations may be explained by the mechanisms involved in the radical antioxidant reactions. Indeed, antioxidant activity of the tested compounds depended on the stressing agent used and the mechanism of action of the antioxidant [56]. Other factors, such as stereo-selectivity of the radicals or the solubility of EOs in different testing systems, may also affect the capacity of individual EO to react and quench different radicals [57]. In this sense, Del Castillo et al. [58] analyzed coffee brews from several roasting processes and reported higher responses by the ABTS test in aqueous *vs.* ethanol dilution. They attributed the difference to the fact that some components, making an important contribution to the antioxidant activity of the aqueous dilutions, were not soluble in ethanol. Probably the fact that the DPPH method was developed in methanol media was responsible for the lower response found. Likewise, Wang and Jiao [59] evaluated the radical scavenging capacity of berry crops using superoxide radicals, hydroxyl radicals, and other reactive oxygen species. The berry crop that had a greater scavenging activity against superoxide radicals did not necessarily have a higher activity to quench hydroxyl radicals [59]. In our study, the major compounds of EO of *O. Basilicum* are alcohol monoterpenes while the major compounds of EO of *L. multiflora* are carbohydrate monoterpenes; this difference of chemical functions of compounds in these two EOs could hence explain the activity differences between the two methods.

**Table 2:** Anti-radical activity of essential oils by DPPH and ABTS methods.

	DPPH	ABTS test
Essential Oils	% Inhibition	μMET/g
*O. basilicum*	55.67±3.38**^A^**	0.69±0.03**^B^**
*O. americanum*	15.90±5.73**^C^**	0.48±0.01**^C^**
*H. spicigera*	41.70±3.10**^A,B^**	0.52±0.02**^C^**
*L. multiflora*	42.23±2.73**^A,B^**	1.02±0.02**^A^**
*A. conyzoides*	32.37±4.25**^B,C^**	0.53±0.02**^C^**
*E. camaldulensis*	43.40±4.13**^A,B^**	not determined
*Z. officinale*	36.10±3.51**^A,B,C^**	0.34±0.03**^D^**
Quercetin	73.13±5.25	8.96±0.07

DPPH, (2,2-diphenyl-1-picrylhydrazyl); ABTS (2,2′-azinobis-[3-ethylbenzothiazoline-6-sulfonic acid]); Values are expressed as mean values ± standard deviation (n = 3 experiments in quadruplicate); DPPH activities is expressed as inhibitory percentage at and ABTS activities are given in mmol TE/g (10^−3^ mol Throlox equivalent/g of extract); Concentrations of the extracts Throlox of 100 µg/mL for DPPH and 1 mg/mL for ABTS used as standard; A, B, C, D: means followed by the same letter are not significantly different (p>0.05).

In addition monoterpenes found in these EOs may act as antioxidant agents. Previous studied showed that oxygenated monoterpenes, such as thymol, carvacrol and α-terpineol, were mainly responsible for the antioxidant potential of the plant oils which contain them [60], [61]. The monoterpene β-caryophyllene also possesses a free radical scavenging activity using the DPPH assay [62].

### Anti-inflammatory properties of the essential oils

Chronic inflammation increases the risk for various cancers, indicating that eliminating inflammation may represent a valid strategy for cancer prevention and therapy. For that purpose we evaluated the anti-inflammatory properties of EOs we isolated. Among the seven plants, *Z. officinale* presented the highest anti-inflammatory activity indeed a 50.9% of inhibition of lipoxygenase at 0.4 mg/ml while this inhibition was complete (100%) at 8 mg/ml (Table 3). *Z. officinale* activity (50.9% of inhibition at 0.4 mg/ml) is highly interesting compared to that of the positive control quercetin (52.32% of inhibition at 0.1 mg/ml). *O. basilicum* (98.2%), *E. camaldulensis* (96.4%), *L multiflora* (96.4%), *H. spicigera* (75.1%), *A. conyzoides* (48.3%) and *O. americanum* (31.6%) (Table 3) were active at 8 mg/ml and no significant effect was observed at 0.4 mg/ml.. These results are in agreement with several studies of anti-inflammatory abilities of sesquiterpenes compounds [63]. Lipoxygenases and their active metabolites (HPETE and HETE) are involved in many human cancers [64]. Pidgeon et al. [65] have shown the involvement of 15-lipoxygenase in the development of breast, prostate, colorectal cancers where it is over expressed [65]. Moreover, the 5-LOX mRNA and/or its activating protein FLAP are over expressed in prostate cancer [66].

**Table 3:** Inhibition percentage of lipoxygenase by essential oils.

Essential oils	* *O. basilicum*	* *O. americanum*	* *H. spicigera*	* *L. multiflora*	* *A. conyzoides*	* *E. camaldulensis*	** *Z. officinale*	**Quercetin (control)
**Inhibition (%)**	98.2±6.1**^C^**	31.6±16.2**^D^**	75.1±10.6**^C^**	96.9±4.0**^C^**	48.3±19.1**^D^**	96.4±3.3**^C^**	50.9±0.2**^B^**	52.3±0.7**^A^**

Values are expressed as mean values ± standard deviation (n = 3 experiments); %, percentage;

*, 8 mg/ml in the reaction medium;

**, 0.4 mg/ml;

***, 100 µg/ml in the reaction medium;

A, B, C, D: means followed by the same letter are not significantly different (p>0.05).

### Anti-proliferative effects of the essential oils

To best of our knowledge, this is the first report on the anti-proliferative activity of these EOs. To test such effects, we chose two types of cancer cell lines. Prostate cancer cell lines, LNCaP and PC3, represent the paradigm of the prostate tumors with androgen sensitivity and androgen resistance, respectively; besides, this cancer has its incidence increasing in Westernized countries. Glioblastoma is recognized as the most common and lethal form of central nervous system cancer. Currently used surgical techniques, chemotherapeutic agents, and radiotherapy strategies have done very little in extending the life expectancies of patients diagnosed with this tumor. SF-767 and SF-763 cell lines are radiotherapy resistant; in SF-767 cells, STAT3 pathway is activated while in SF-763, both STAT3 and AKT pathways are constitutively activated.

**Figure 3:**
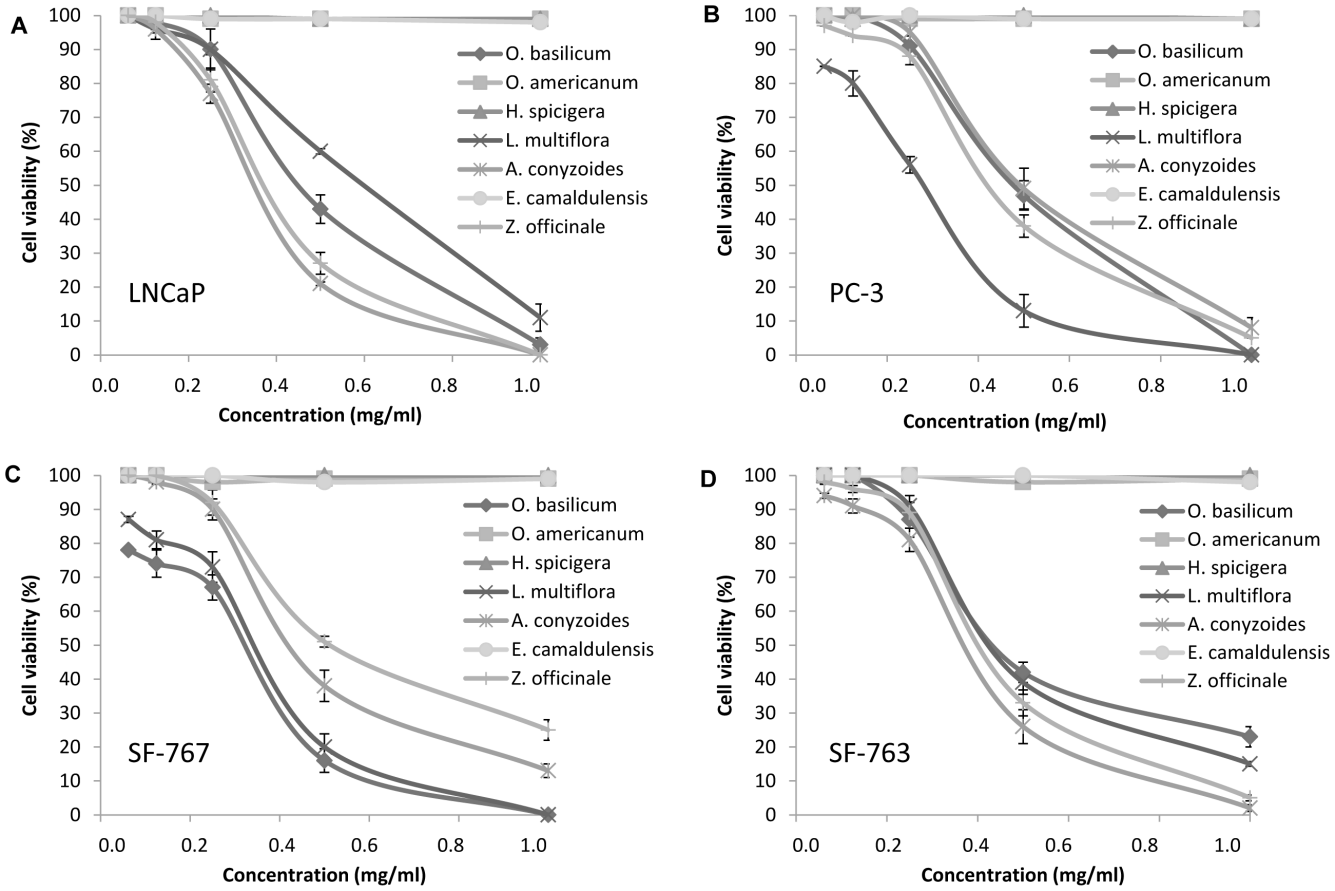
Dose-dependent anti-proliferative activity of EOs after 72 hours of exposure. A LNCaP cells; B) PC-3 cells; C) SF-767 cells; D) SF-763 cells. Experiments were performed 3 times in octuplets.

**Figure 4:**
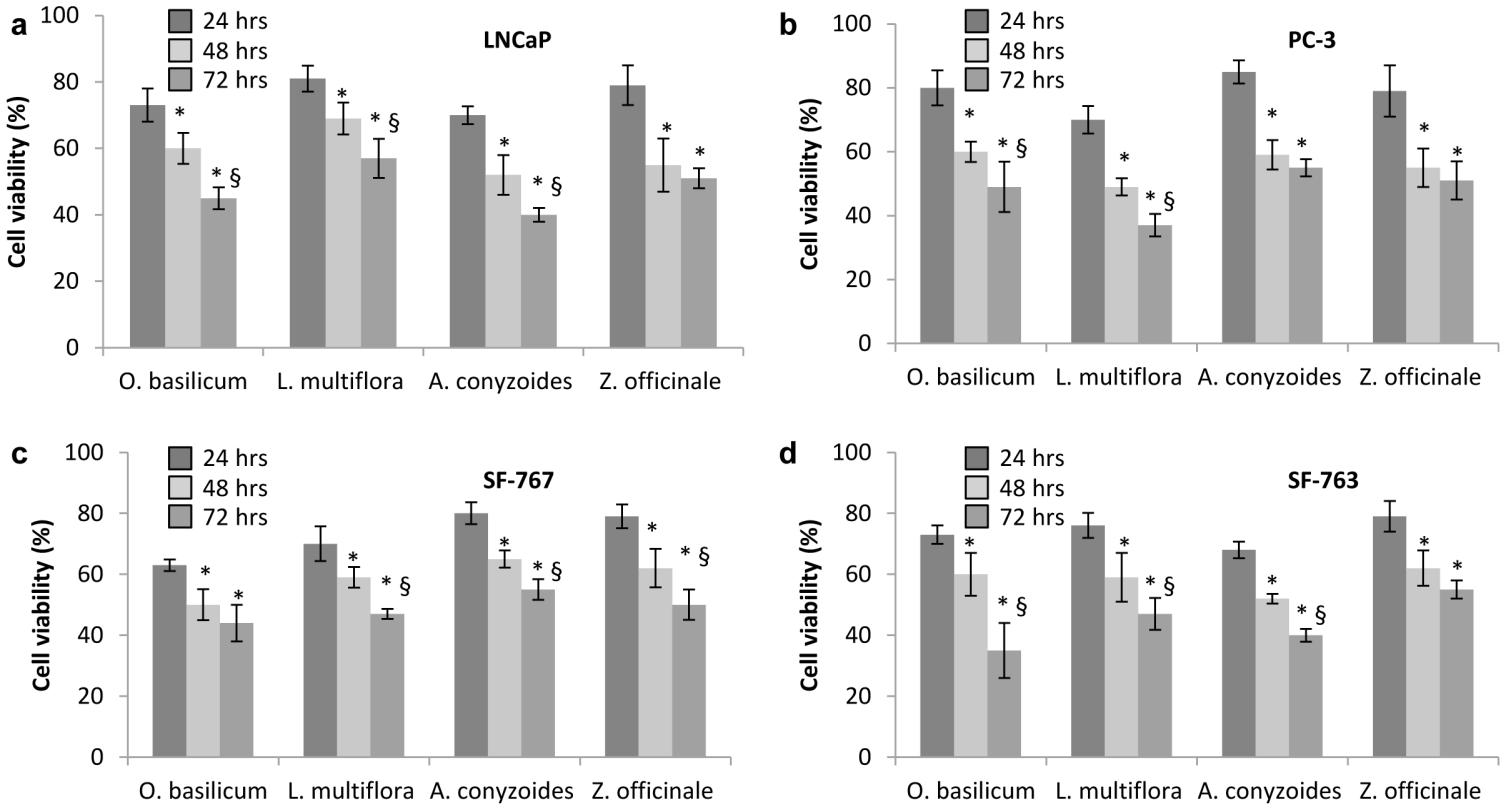
Time-dependent anti-proliferative activity of EOs after 24, 48 and 72 hours of exposure. Cells were incubated at IC_50_ of each EO. a, LNCaP cells; b, PC-3 cells; c, SF-767 cells; d, SF-763 cells. *, p<0.05 compared to 24 hrs of treatment; §, p<0.05 compared to 48 hrs of treatment. Experiments were performed 3 times in octuplets.

*O. basilicum*, *Z. officinale*, *L. multiflora* and *A. conyzoides* have shown anti-proliferative activity on both LNCaP and PC-3 cell lines of prostate cancer and both SF-767 and SF-763 cell lines of glioblastoma (Table 4). Conversely, EOs from *E. camaldulensis*, *O. americanum* and *H. spicigera* did not present any antiproliferative activity even at a maximum concentration of 8 mg/ml (Table 4). *A. conyzoides* (IC_50_ 0.35 mg/mL) and *Z. officinale* (IC_50_ 0.38 mg/mL) have the highest antiproliferative activity on LNCaP cell line. EOs of L. multiflora and *O. basilicum* were the most active on PC-3 cell line (IC_50_ 0.30 mg/mL) and SF-767 cell line with (IC_50_ 0.30 mg/mL). Monoterpenes prevent the carcinogenesis process at both initiation and promotion/progression stages [67]. Monoterpenes are effective in treating early and advanced cancers. In fact, monoterpene pirillyl alcohol has been described to have an anti-proliferative activity on glioblastoma by inhibiting the Na/K-ATPase pump [68]. Other monoterpenes such as limonene have been shown to prevent mammary, liver, lung, and other cancers [67]. Thus, the high content of monoterpenes of these essential oils could explain their antiproliferative activity. In our study, all anti-proliferative effects of the tested EOs are dose- (Figure 3) and time- (Figure 4) dependent.

**Table 4:** IC_50_ (mg/ml) of essential oils tested on LNCaP and PC-3 prostate cancer cell lines, and SF-767 and SF-763 glioblastoma cell lines.

	Prostate cancer		Glioblastoma	
Essential oils	LNCaP	PC-3	SF-767	SF-763
***O. basilicum***	0.46±0.11**^A,B^**	0.45±0.07**^B^**	0.30±0.05**^A^**	0.43±0.10**^A^**
***O. americanum***	not active	not active	not active	not active
***H. spicigera***	not active	not active	not active	not active
***L. multiflora***	0.58±0.14**^B^**	0.30±0.03**^A^**	0.31±0.02**^A,B^**	0.47±0.14**^A^**
***A. conyzoides***	0.35±0.03**^A^**	0.49±0.08**^B^**	0.43±0.09**^B,C^**	0.38±0.06**^A^**
***E. camaldulensis***	not active	not active	not active	not active
***Z. officinale***	0.38±0.11**^A^**	0.42±0.05**^B^**	0.48±0.09**^C^**	0.44±0.08**^A^**

IC_50_, Inhibiting Concentration 50; Values are expressed as mean values ±standard deviation (n = 3 experiments in octuplets); value with same letter within each column could be considered as identical (p>0.05).

All EOs with an anti-proliferative activity (Table 4) are also antioxidant (Table 2) and show anti-inflammatory properties (Table 3). Even though there is a relationship between these three activities, the various mechanisms involved for each EO could explain why the variation of these effects. Indeed reactive oxygen species may interact with and modify cellular protein, lipid, and DNA, which results in altered target cell function. The accumulation of oxidative damage has been implicated in both acute and chronic cell injury including possible participation in the formation of cancer [69]. A link between inflammation and cancer has long been suspected, but its molecular nature remained still to be defined [70]. Chronic infection and consecutive inflammation may directly affect the cells that eventually become transformed as well as exert indirect effects on the tumor cell through surrounding cells [70].

**Figure 5:**
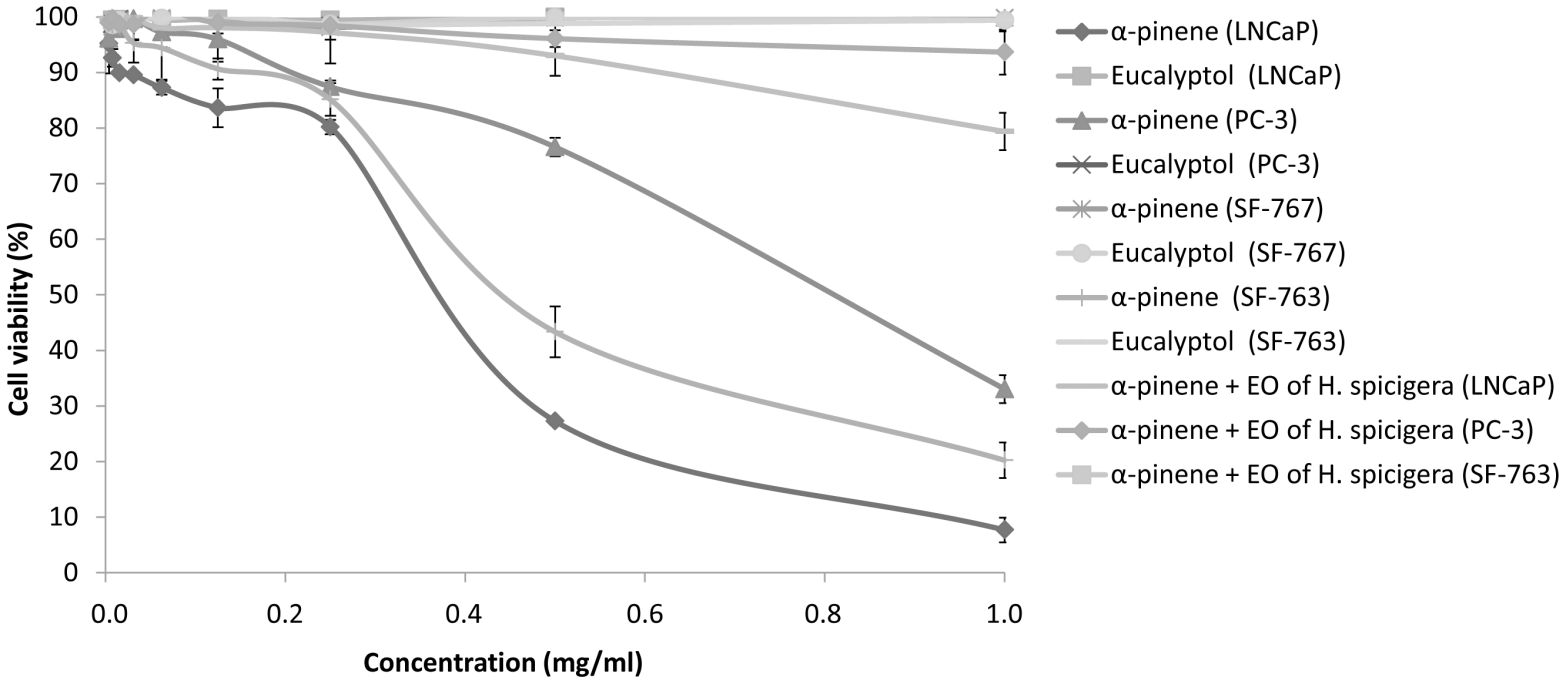
Dose-dependent anti-proliferative activity of purified compounds and their combination with essential oils after 72 hours of exposure on LNCaP and PC-3 cell lines of prostate cancer, and on SF-767 and SF-763 cell lines of glioblastoma. Experiments were performed 3 times in octuplets.

EOs of *O. americanum*, *H. spicigera* and *E. camaldulensis* have shown antioxidant activity and anti-inflammatory activity but no anti-proliferative activity. The Eucalyptol or 1, 8-cineol, the major compound of essential oil of *O. americanum* and *E. camaldulensis* has also not effect on LNCaP and PC-3 cell lines of prostate cancer, and the SF-767 and 763 cell lines of glioblastoma to a maximum of 1 mg/mL concentration tested (Figure 5). However, Murata et al. showed that 1, 8-cineole induced specific apoptosis, not necrosis, in human colon cancer cell lines HCT116 and RKO [71]. Each type of cancer involves a specific signaling pathway. Moreover an anticancer compound does not necessarily treat all types of cancer; such is the case of α-pinene (Figure 5). What may explain the fact that the 1, 8 cineole is inactive on prostate cancer and glioblastoma. Accordingly the results of our work show that essential oils *O. americanum*, *H. spicigera* and *E. camaldulensis* are ineffective against prostate cancer and glioblastoma.

α-pinene, the major compound of *H. spicigera*, showed anti-proliferative activity on LNCaP and PC-3 cell lines of prostate cancer with IC_50_ of 0.37 mg/ml and 0.82 mg/ml, respectively. The combination of α-pinene and the essential oil of *H. spicigera* (50∶50, v∶v) resulted in a decreased of this activity (Figure 5). This significant decrease in the activity of combinations of α-pinene with EO of *H. spicigera* leads to an antagonistic effect between α-pinene and the essential oil of *H. spicigera*. This antagonistic effect could justify the inactivity of EO from *H. spicigera* alone (Figure 3). On SF-763 cells IC_50_ of α-pinene is 0.43 mg/ml while no significant anti-proliferative activity was observed on SF-767 cells (Figure 5). The main difference between these two cell lines regards the survival pathways: indeed STAT3 pathway is activated in SF-767 cells, while both Akt and STAT3 pathways are induced in SF-763 cells. These differences were hypothesized to explain chemoresistance [72] and could also be linked to the differential response to α-pinene.

In conclusion we have evaluated for the first time the chemical composition, ability to scavenge free radicals, anti-inflammatory activity by inhibition of lipoxygenase, and anti-proliferative activity on various cancer cell lines of EOs from plants of Burkina Faso. EOs enriched in sequiterpenes presented the highest anti-inflammatory activity while those enriched in monoterpenes and oxygenated terpenes showed highest anti-proliferative characteristics. This work therefore provides a scientific basis for the local use of these plants and also a tool promoting the use therapeutic benefits of EOs from Burkina Faso.
